# PifC and Osa, Plasmid Weapons against Rival Conjugative Coupling Proteins

**DOI:** 10.3389/fmicb.2017.02260

**Published:** 2017-11-16

**Authors:** María Getino, Carolina Palencia-Gándara, M. Pilar Garcillán-Barcia, Fernando de la Cruz

**Affiliations:** Instituto de Biomedicina y Biotecnología de Cantabria, Universidad de Cantabria—Consejo Superior de Investigaciones Científicas, Santander, Spain

**Keywords:** fertility inhibition, antimicrobial resistance, bacterial conjugation, plasmid, PifC protein, Osa protein, type IV coupling protein, mobilization

## Abstract

Bacteria display a variety of mechanisms to control plasmid conjugation. Among them, fertility inhibition (FI) systems prevent conjugation of co-resident plasmids within donor cells. Analysis of the mechanisms of inhibition between conjugative plasmids could provide new alternatives to fight antibiotic resistance dissemination. In this work, inhibition of conjugation of broad host range IncW plasmids was analyzed in the presence of a set of co-resident plasmids. Strong FI systems against plasmid R388 conjugation were found in IncF/MOB_F12_ as well as in IncI/MOB_P12_ plasmids, represented by plasmids F and R64, respectively. In both cases, the responsible gene was *pifC*, known also to be involved in FI of IncP plasmids and *Agrobacterium* T-DNA transfer to plant cells. It was also discovered that the R388 gene *osa*, which affects T-DNA transfer, also prevented conjugation of IncP-1/MOB_P11_ plasmids represented by plasmids RP4 and R751. Conjugation experiments of different mobilizable plasmids, helped by either FI-susceptible or FI-resistant transfer systems, demonstrated that the conjugative component affected by both PifC and Osa was the type IV conjugative coupling protein. In addition, *in silico* analysis of FI proteins suggests that they represent recent acquisitions of conjugative plasmids, i.e., are not shared by members of the same plasmid species. This implies that FI are rapidly-moving accessory genes, possibly acting on evolutionary fights between plasmids for the colonization of specific hosts.

## Introduction

Bacteria display a series of mechanisms to control conjugative DNA transfer, as they do for any other physiological process (Getino and de la Cruz, 2017). If properly manipulated, these mechanisms may be useful to prevent dissemination of antibiotic resistance determinants, which are mainly transferred by conjugation (Waters, 1999; Norman et al., 2009). Natural means to inhibit conjugation can be encoded by recipient bacteria as defense systems against potentially harmful invading genomes, as are the cases of restriction-modification (Wilkins, 2002) or CRISPR-Cas systems (Marraffini and Sontheimer, 2008). Mechanisms to control conjugative transfer are also present in plasmids themselves. For example, all conjugative plasmids code for exclusion systems that act in recipient bacteria to prevent competition between identical plasmid backbones, block uneconomical excess of conjugative transfer, and protect recipients from lethal zygosis (Garcillan-Barcia and de la Cruz, 2008).

FI systems are best known as regulators of plasmid transfer in donor bacteria. As such, they minimize the burden associated with constitutive expression of the conjugative machinery as well as minimizing phage vulnerability. This is the case of the FinOP system of IncF plasmids (Frost and Koraimann, 2010). Since the relevant products act *in trans*, the FinOP system collaterally inhibits conjugation of related plasmids (Frost and Koraimann, 2010). Beyond FinOP, there exist “unusual” FI systems that affect conjugation of unrelated co-resident plasmids. Their mechanisms and functional roles are different, and poorly known. For instance, they could represent competition factors for colonization of new hosts (Gasson and Willetts, 1975, 1977). Among them, different plasmids (IncI1, IncFI, CloDF13-like, and IncX) inhibit the transfer of IncF plasmids, although the responsible genes were not characterized (Gasson and Willetts, 1975). Two genes of IncP-1α plasmids, *fiwA* and *fiwB*, block conjugation of IncW plasmids (Fong and Stanisich, 1989). Genes *fipA, pifC*, and *tir*, encoded by IncN, IncFI, and IncFII plasmids, inhibit the fertility of IncP plasmids (Miller et al., 1985; Tanimoto et al., 1985; Winans and Walker, 1985). The best studied FI system, *osa* of IncW plasmids, inhibits transfer of *Agrobacterium tumefaciens* T-DNA encoded in pTi plasmid to plant cells (Close and Kado, 1991; Maindola et al., 2014).

Understanding the interaction network between transmissible plasmids is essential to know how the main carriers of antibiotic resistance genes disseminate in the environment. In this work, we discovered two novel plasmid interactions by analyzing transfer of broad host range, MOB_F11_/IncW plasmids in the presence of a representative set of conjugative plasmids. We found that genes *pifC* (from IncFI or IncI1 plasmids) and *osa* (from IncW plasmids) prevent conjugation of IncW and IncP plasmids, respectively, by targeting their coupling proteins. This could explain why these plasmid groups are not prevalent in *E. coli*, in which IncF and IncI plasmids prevail (de Toro et al., 2014).

## Materials and methods

### Bacterial strains and plasmids

Derivatives of strain *E. coli* DH5α (Grant et al., 1990), containing different combinations of conjugative and/or mobilizable plasmids were used as donors in mating experiments (Table 1). A rifampicin-resistant derivative of *E. coli* MDS42 (Posfai et al., 2006) was used as the recipient strain. MOB_F11_ conjugative plasmids were R388 (Datta and Hedges, 1972), pIE321 (Gotz et al., 1996), pMBUI4 (Brown et al., 2013), R7K (Coetzee et al., 1972), or R388Δ*kfrA-osa* (del Campo, 2016). Mobilizable plasmids used were the kanamycin-resistance derivatives of ColE1 (Van Rensburg and Hugo, 1969), and RSF1010 (Derbyshire et al., 1987), and the ampicillin-resistance derivative of CloDF13 (van Putten et al., 1987). A list of co-resident plasmids can be found in Table 1.

**Table 1:** Fertility interactions of IncW plasmids with a representative set of compatible co-resident plasmids.

**Co-resident plasmid**	**Inc**	**MOB**	**MPF**	**References**	**IncW plasmid conjugation**	**Co-resident conjugation**
pKM101	IncN	F11	T	Langer et al., 1981	3.4^**^	0.7
pOX38::*CmR*	IncFI	F12	F	Chandler and Galas, 1983	7 · 10^−5^^***^	0.8
R1*drd-19*	IncFII	F12	F	Meynell and Datta, 1967	1.0	1.4
R100-1	IncFII	F12	F	Yoshioka et al., 1987	0.8	0.9
pRL443	IncP-1α	P11	T	Elhai et al., 1997	4 · 10^−4^^***^	8 · 10^−2^^***^
R751	IncP-1β	P11	T	Thorsted et al., 1998	2.4	8 · 10^−3^^***^
R64*drd11*	IncI1α	P12	I	Komano et al., 1990	8 · 10^−4^^***^	3.1
pCTX-M3	IncL/M	P131	I	Golebiewski et al., 2007	0.4	0.9
pOLA52	IncX1	P3	T	Sorensen et al., 2003	1.0	1.3
R6K	IncX2	P3	T	Kolter and Helinski, 1978	7 · 10^−4^^***^	0.4
drR27	IncHI1	H11	F	Whelan et al., 1994	0.8	0.9

Conjugation of IncW and different plasmids when co-resident in donor bacteria. The result shows the mean of conjugation frequencies obtained from at least four independent experiments normalized to that in the absence of co-resident plasmids (

** *p < 0.01*,

*** *p < 0.001). IncW plasmid R388 was used in all matings, except when Tp^R^ plasmids pCTX-M3 or R751 were co-resident. In such case, IncW plasmid pIE321 was used. Inc, incompatibility group (Taylor et al., 2004). MOB, MOB relaxase group (Garcillan-Barcia et al., 2009). MPF, mating pair formation type (Guglielmini et al., 2011)*.

### Reagents

When appropriate, antibiotics (Apollo) were added at the following concentrations: ampicillin (Ap; 100 μg/ml), chloramphenicol (Cm; 25 μg/ml), kanamycin (Km; 50 μg/ml), nalidixic acid (Nx; 20 μg/ml), rifampicin (Rif; 50 μg/ml), tetracycline (Tc; 10 μg/ml), and trimethoprim (Tp; 20 μg/ml). Arabinose (Sigma-Aldrich) 0.001% (w/v) was used as transcription inducer. Bacterial cultures were set up in LB-broth and LB-agar (Pronadisa). M9-salts (Sigma-Aldrich) were used to resuspend bacteria after mating and to perform serial dilutions.

### Construction of pBAD33::*trwB*, pBAD33::*pifC*, and pBAD33::*osa*

Coupling protein gene *trwB* from plasmid R388 (Datta and Hedges, 1972) was amplified by PCR using primers F*Kpn*ITrwB and R*Hin*dIIITrwB. Gene *pifC* from plasmid pOX38 (Chandler and Galas, 1983) was amplified using primers F*Kpn*IPifC and R*Hin*dIIIPifC. Gene *osa* from plasmid R388 (Datta and Hedges, 1972) was amplified using primers F*Kpn*IOsa and R*Hin*dIIIOsa (Supplementary Table 1). These primers introduced *Kpn*I and *Hin*dIII sites at one and another end of the respective amplicon. Each amplicon, and the pBAD33 vector (Guzman et al., 1995), were digested with *Kpn*I and *Hin*dIII endonucleases (Thermo-Fisher). Final constructs pBAD33::*trwB*, pBAD33::*pifC*, and pBAD33::*osa* were obtained after ligation, using T4 DNA ligase (Thermo-Fisher), and electroporation into *E. coli* DH5α competent cells.

### Conjugation assays

Donor and recipient cultures in stationary phase were washed in LB-broth and mixed in a 1:1 donor-recipient ratio. Then, a 200 μl mix was centrifuged and resuspended in 15 μl LB-broth. 5 μl of this mixture were placed on top of 96-well microtiter plate wells containing 150 μl LB-agar and conjugation was allowed to proceed for 1 h at 37°C. Conjugation of a derepressed R27 derivative, drR27, was performed for 2 h at 25°C. In all cases, bacteria were then resuspended in 150 μl M9-salts and corresponding dilutions plated on selective media. Conjugation frequencies were estimated as the number of transconjugant cells per donor (T/D) and means were calculated using decimal logarithms of data.

### Statistical analysis

Comparison of the means between two different conditions was carried out by using *t-*test tool from GraphPad Prism^®^ (v 5.0) biostatistics software (San Diego, CA).

### Identification of FI proteins

Psi-blast searches, using proteins Osa_R388 (GenBank Acc. No. FAA00056.1), FiwA_RP4 (CAJ85704.1), FiwB components KlaA_RP4 (CAJ85667.1), KlaB_RP4 (CAJ85666.1), and KlaC_RP4 (CAJ85665.1), PifC protein RepC_F (NP_061420.1), FinC protein OrfD_CloDF13 (NP_052375.1), FinQ_R621a (YP_004823737.1), and FipA_pKM101 (AAC63100.1) as queries, were carried out against the 6,878 plasmid set available at NCBI database in May 12th, 2016 (ftp://ftp.ncbi.nlm.nih.gov/refseq/release/plasmid/). Hits (threshold *e*-value 0.001) were aligned with MUSCLE using default parameters (Edgar, 2004). HMMER 3.0 (Eddy, 2011) was used to build hidden Markov model (HMM) profiles from the alignments, which were in turn used in HMM searches against the plasmid database. For the plasmids encoding the retrieved hits, relaxase MOB families were defined as described in Garcillan-Barcia et al. (2009) and Guglielmini et al. (2011), and mating pair formation types (MPF) were defined as in Guglielmini et al. (2014) and (http://conjdb.web.pasteur.fr/).

## Results

### IncW plasmid conjugation is repressed by IncFI, IncI1, IncP-1α, and IncX2 plasmids

Transfer of IncW plasmids was systematically analyzed in the presence of a representative set of conjugative plasmids in donor bacteria. Depending on antibiotic resistance, either plasmid R388 or pIE321 were tested as prototype IncW conjugative systems. R388 was substituted by pIE321, with a backbone genome 97% identical to R388 (Revilla et al., 2008), when the co-resident plasmid encoded trimethoprim resistance (as in the cases of R751 and pCTX-M3). Conjugation frequencies for each IncW conjugative system and co-resident plasmid were normalized to the mean value of the tested system in the absence of any co-resident plasmid. The results obtained are summarized in Table 1. As shown in the Table, R388 conjugation was significantly affected by the presence of four different plasmids. Specifically, it decreased by 4 logs in the presence of either IncFI plasmid pOX38::*CmR* (F derivative), IncI1 plasmid R64*drd11*, IncP-1α plasmid pRL443 (a kanamycin-sensitive derivative of plasmid RP4), or IncX2 plasmid R6K. Except for the IncN plasmid pKM101, which caused a 3-fold increase in R388 conjugation, the other co-resident plasmids tested (IncFII, IncP-1β, IncX1, IncL/M, and IncHI1) produced no significant change in R388 or pIE321 conjugation. While FI of R388 by IncP-1α plasmids was known to be caused by *fiwA* and *fiwB* genes (Fong and Stanisich, 1989) and R6K inhibited R388 transfer by a reported but unidentified mechanism (Olsen and Shipley, 1975), FI caused by IncFI and IncI1 plasmids had not been reported before.

In turn, IncW plasmids affected the transfer of co-resident IncP-1 plasmids pRL443 and R751. A 2-log reduction was observed on R751 conjugation and 1-log reduction on pRL443 transfer, paralleling the previously observed effect of R388 on transfer of IncP-1 plasmid RP1 (Olsen and Shipley, 1975).

### *pifC* is responsible for both IncP and IncW plasmids FI

**Figure 1:**
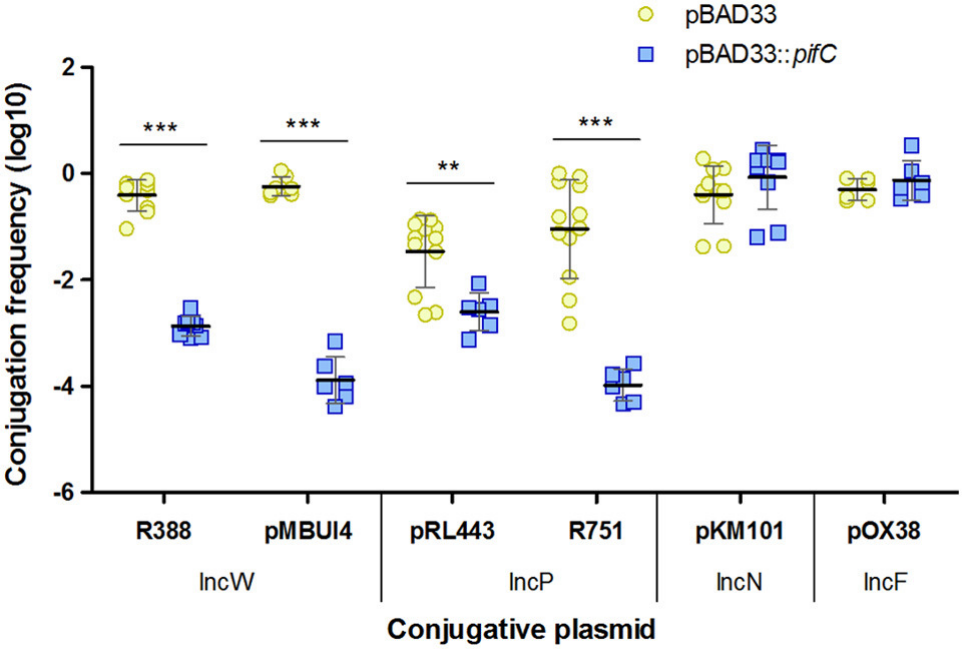
Effect of *pifC* on plasmid conjugative transfer. The conjugation frequencies of plasmids R388, pMBUI4, pRL443, R751, pKM101, and pOX38 in the presence (blue squares) or absence (yellow circles) of *pifC* in donor cells are shown. Each point represents the conjugation frequency (T/D) obtained in one independent experiment. Horizontal and vertical bars represent the mean ± *SD* obtained for each group of data (^**^p < 0.01, ^***^p < 0.001).

The F plasmid was previously described to inhibit the fertility of plasmid RP4 through its gene *pifC* (Miller et al., 1985). Gene *pifC* is present also in the F reduced version pOX38. A 99% identical *pifC* homolog is present in R64*drd11*, an IncI1 plasmid that also affects RP4 conjugation (Datta et al., 1971). The fact that *pifC*-containing plasmids pOX38 and R64*drd11* inhibited conjugation of RP4 and R388, positioned *pifC* as a potential responsible gene in all cases. To prove this fact, *pifC* from pOX38 was cloned in vector pBAD33, giving rise to plasmid pBAD33::*pifC*. Then, conjugation of donor cells containing either IncW, IncP-1, IncN, or IncF conjugative plasmids was tested in the presence or absence of pBAD33::*pifC*. As shown in Figure 1, conjugation of the IncW prototype plasmid R388 and the IncW-like plasmid pMBUI4 were significantly affected by *pifC* expression in donors. This result was comparable to that exerted over the IncP-1 plasmids and indicated that *pifC* is actually the gene responsible for IncW conjugation inhibition by IncFI and IncI1 plasmids. However, the impact on R388 fertility of *pifC* expressed from pBAD33::*pifC* was unexpectedly weaker than the impact of the complete F plasmid, suggesting that one or more additional F loci might be involved in inhibiting R388 transfer. In addition, PifC had no effect on the transfer frequency of the IncN plasmid pKM101, and *pifC* overexpression did not affect pOX38 conjugation.

### IncW and IncP coupling proteins are the targets of *pifC* inhibition

The R388 origin of transfer, TrwA accessory protein, TrwB coupling protein, and TrwC relaxase comprise the R388 MOB genetic module, involved in conjugative DNA processing prior to plasmid transfer. Among these components, previous work pointed to TrwB as the target of various FI systems. This was the case of FipA of pKM101 and PifC of F, which inhibited RP4 conjugation by affecting TraG coupling protein (Santini and Stanisich, 1998), and Osa of pSa, which prevented T-DNA transfer to plant cells by limiting substrate binding to VirD4 coupling protein (Cascales et al., 2005).

**Figure 2:**
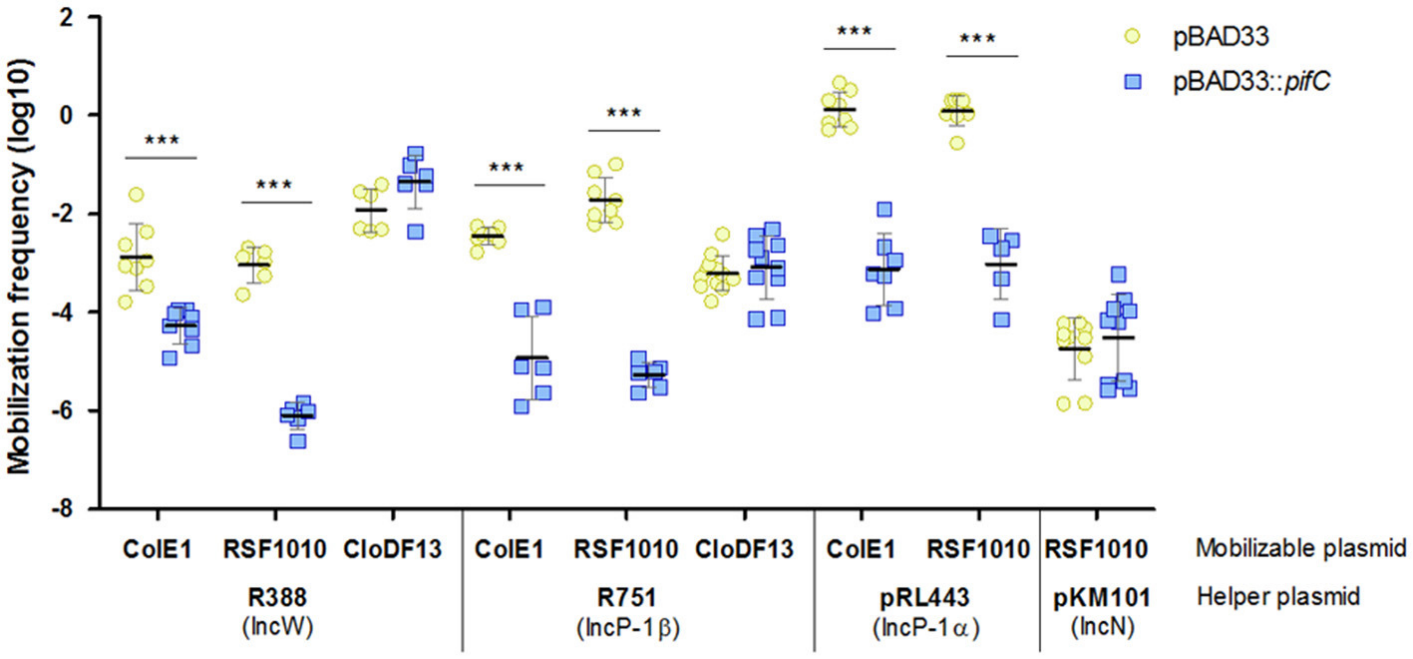
Effect of *pifC* on the transfer of mobilizable plasmids by representative conjugative helpers. The mobilization frequencies of ColE1, RSF1010, and CloDF13, using either R388, R751, pRL443, or pKM101 as helpers, in the presence (blue squares) or absence (yellow circles) of *pifC* in donor cells, are shown. Each point represents the mobilization frequency (T/D) of one independent experiment. Horizontal and vertical bars represent the mean ± *SD* obtained for each group of data (^***^p < 0.001).

To demonstrate that TrwB was also targeted by PifC, we analyzed transfer of three mobilizable plasmids with different requirements in the presence and absence of *pifC*. For mobilization, R388 provided the MPF genes to construct the mating channel that connects donors with recipients. Plasmids ColE1 and RSF1010 need R388 coupling protein as well as the MPF genes for mobilization, whereas CloDF13 only needs R388 MPF because it encodes its own coupling protein (Cabezon et al., 1994). The results of these experiments are shown in Figure 2. While CloDF13 mobilization was not affected by *pifC*, transfer of ColE1 and RSF1010 plasmids was inhibited when expressing *pifC*. The absence of effect in CloDF13 mobilization discarded R388 MPF components as potential targets, confirming our previous hypothesis. The inhibitory effect of *pifC* in ColE1 and RSF1010 mobilization suggests that R388 coupling protein is the most likely target of this FI system. The other, more unlikely, option is that *oriT*, relaxase, or accessory proteins of R388, ColE1, and RSF1010 were independently affected by *pifC*. However, previous experiments showing high mobilization frequencies of ColE1 by either F and R64 helper plasmids (Cabezon et al., 1997), both encoding *pifC*, indicate that ColE1 mobilization machinery is not affected by *pifC*. To reinforce this data, RSF1010 plasmid was mobilized by the *pifC*-unaffected conjugative plasmid pKM101. The absence of effect when employing pKM101 coupling protein indicates that R388 coupling protein was the actual target of *pifC* inhibition. As controls, we included IncP plasmids R751 and pRL443 as helpers of ColE1, RSF1010, and CloDF13 mobilization, which behaved similarly to R388.

**Figure 3:**
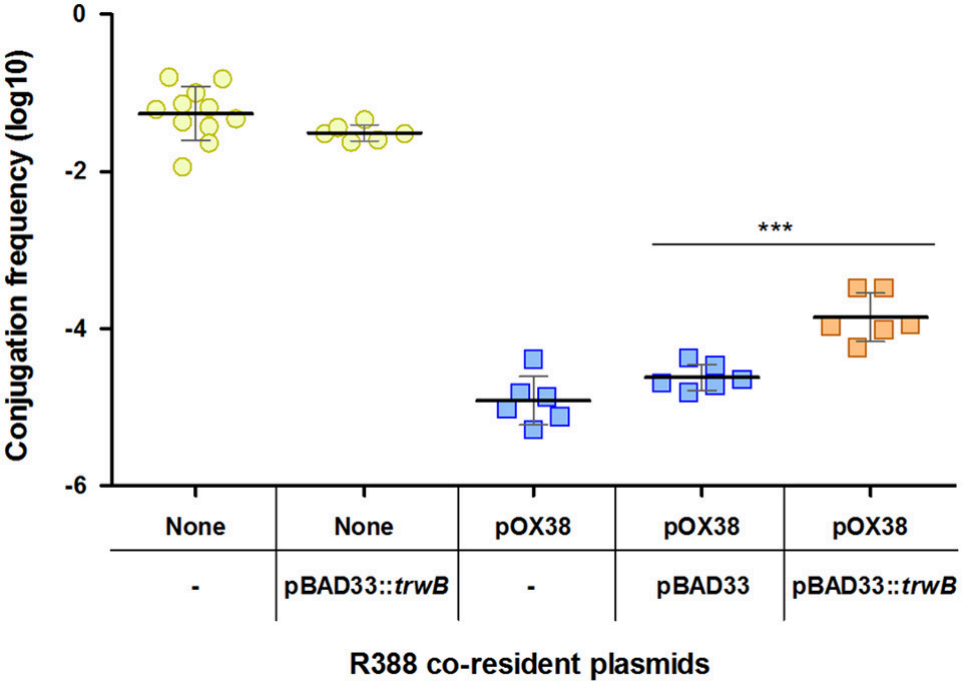
Modulation of the PifC-mediated FI of R388 by overexpression of the coupling protein. Each point represents the conjugation frequency (T/D) of one independent experiment, in the presence or absence of pOX38 and R388 coupling protein TrwB. Horizontal and vertical bars represent the mean ± *SD* obtained for each group of data (^***^p < 0.001). None/- (indicated by yellow circles), R388 alone. None/pBAD33::*trwB* (yellow circles), R388 in the presence of pBAD33::*trwB*. pOX38/- (blue squares), R388 in the presence of pOX38::*KmR*. pOX38/pBAD33 (blue squares), R388 in the presence of pOX38::*KmR* and pBAD33 empty vector. pOX38/pBAD33::*trwB* (orange squares), R388 in the presence of pOX38::*KmR* and pBAD33::*trwB*.

With the aim of improving R388 transfer efficiency in the presence of plasmid pOX38, we overexpressed TrwB coupling protein in donor cells. As observed in Figure 3, increased expression of *trwB* partially relieves inhibition, the frequency of transfer increasing 6-fold when compared to the frequency in the presence of basal *trwB*. As a control, in the absence of pOX38, *trwB* overexpression did not produce a significant change in R388 conjugation. In addition, the empty expression vector pBAD33 did not cause any impact in pOX38 inhibitory effect. Overall, the significant alleviation of R388 transfer by *trwB* overexpression indicates that the activity of the coupling protein is affected by PifC.

### R388 *osa* is responsible for IncP FI

**Figure 4:**
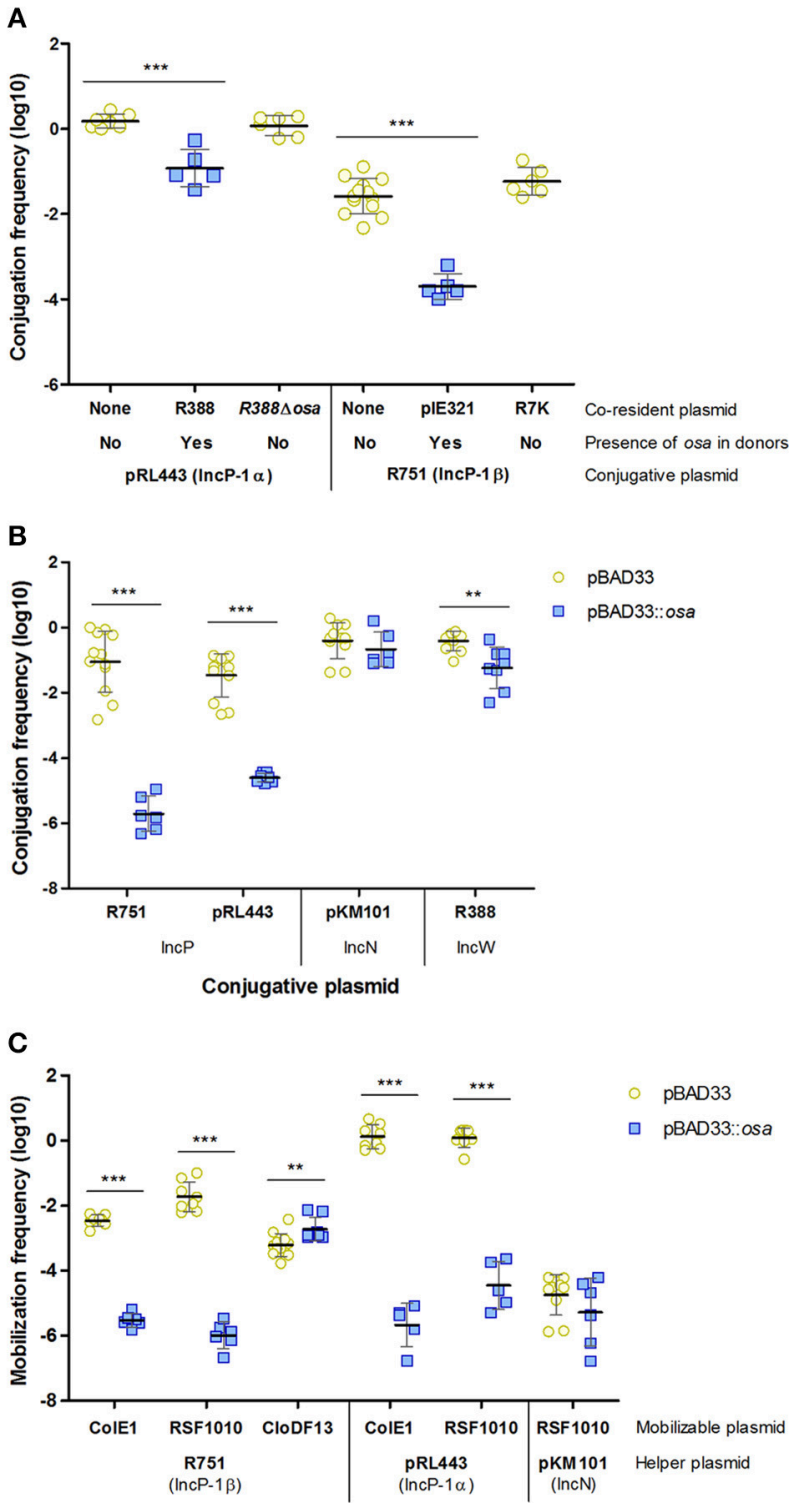
Effect of *osa* on the transfer of conjugative and mobilizable plasmids. **(A)** FI of IncP-1 plasmids mediated by IncW plasmids. **(B)** FI of IncP-1 plasmids mediated by *osa*. **(C)** Effect of *osa* on the transfer frequency of mobilizable plasmids by IncP-1 helpers. Each point represents the transfer frequency (T/D) of one independent experiment in the presence (blue squares) or absence (yellow circles) of *osa* in donor cells. Horizontal and vertical bars represent the mean ± *SD* obtained for each group of data (^**^p < 0.01, ^***^p < 0.001).

As shown in Table 1, IncW plasmids prevented conjugation of IncP plasmids pRL443 and R751. Although a similar effect was previously found (Olsen and Shipley, 1975), the responsible gene was not identified. Most IncW plasmids encode *osa*, a FI function affecting transfer of *A. tumefaciens* T-DNA to plant cells (Close and Kado, 1991). Plasmids of the IncW family that lack this gene, such as R7K (Coetzee et al., 1972) or the R388 derivative R388Δ*kfrA-osa* (del Campo, 2016), when used as co-residents with pRL443 and R751, resulted in conjugation frequencies as high as in donors containing just the IncP plasmids (Figure 4A). This result indicates that Osa is the FI function affecting IncP plasmids. To confirm this hypothesis, we cloned *osa* of R388 in the pBAD33 vector and expressed it in donor cells. As observed in Figure 4B, conjugation of IncP plasmids was inhibited when *osa* was expressed in donor cells. In this case, as expected, the FI effect is stronger when *osa* is expressed from pBAD33::*osa* instead of R388 plasmid. On the other hand, *osa* did not affect transfer of the IncN plasmid pKM101. It is worth to mention that conjugation of the *osa*^+^ IncW plasmid R388 was slightly affected by *osa* overexpression in donor cells. This inhibition could be related to the fact that *osa* and *fiwA* are homologs and thus, *osa* overexpression could mimic FiwA activity to some extent.

### IncP coupling protein is the target of Osa FI

In order to check if the molecular target of Osa was the IncP coupling protein, we analyzed the mobilization of plasmids with and without their own coupling protein (CloDF13 and ColE1/RSF1010, respectively), using affected and unaffected helper plasmids (pRL443/R751 and pKM101, respectively) in the presence of *osa*. The results obtained are shown in Figure 4C. While mobilization of ColE1 and RSF1010 by IncP plasmids pRL443 and R751 was inhibited by *osa*, CloDF13 transfer was resistant to the inhibitory effect. These results suggested that TraG, the coupling protein of IncP plasmids, was the molecular target of Osa. The absence of effect when RSF1010 was mobilized by the unaffected plasmid pKM101 discarded other MOB components as potential targets, confirming our hypothesis.

### Distribution and abundance of FI factors

**Figure 5:**
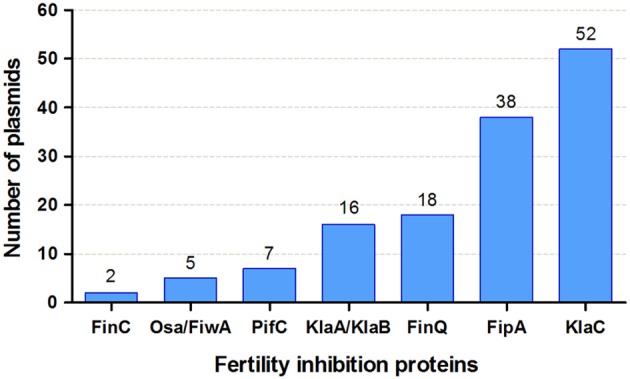
Abundance of FI protein families in the NCBI plasmid database. The figure shows the number of plasmids that contain one hit to each of the FI protein families. See details in section Materials and Methods and Supplementary Table 2.

From the FI genes named and reported in the literature, the gene sequence is available for just a few, namely those encoding factors Osa, FiwA, FiwB (KlaA, KlaB, and KlaC proteins), PifC, FinC, FinQ, and FipA. To assess their abundance and distribution in different plasmid families, we searched for homologs of these FI proteins in NCBI plasmid database. At least one protein with homology to one of the protein families was found for 129 out of the 6,878 plasmids contained in the database (Supplementary Table 2). Although FI was studied mainly in plasmids from enterobacteria and agrobacteria, other bacterial classes are represented in the list. The abundance of each protein is shown in Figure 5. KlaC and FipA were the most abundant. Profile-profile comparisons showed that Osa and FiwA were homologs, as well as KlaA and KlaB. The remaining protein families showed unrelated HMM profiles.

The FiwB-mediated FI phenotype exhibited by IncP-1α on IncW plasmid transfer requires three proteins, KlaA, KlaB, and KlaC (Goncharoff et al., 1991). Only 7 out of the 52 *klaC*-encoding plasmids also encoded *klaA* and *klaB* genes. In turn, nine plasmids lacking *klaC* but encoding other *fiwB* genes were found (three contained KlaA and KlaB, while six KlaA or KlaB). Although FiwB was described and studied in an IncP-1α/MOB_P_/MPF_T_ plasmid, it is absent from other IncP-1 groups and is more widely found in different replicons containing a MOB_H_/MPF_F_ transfer system (6 out of the 7 *klaABC*-encoding plasmids).

Each FI family comprises homologs retrieved from both, highly related backbones and plasmids from different replication, MOB and MPF groups. The exception was the FinC family, which comprises only two members encoded in highly-similar mobilizable backbones. Within each protein family, the closest homologs were carried by highly-identical plasmid backbones (i.e., members of the same Inc group). Plasmids of the same replication group tend to encode the same type of FI factor (e.g., FipA in IncN plasmids). Nevertheless, plasmid membership to an Inc group does not necessarily anticipate the presence of a common FI factor in all members. Conserved backbones can encode different factors (e.g., some IncI1 plasmids encode *pifC*, while others encode *finQ*; *fiwA* is present in the α subgroup of IncP-1 plasmids, but is absent in the rest of subdivisions).

Plasmids tend to encode a single FI factor. The exception, pKPC-LKEc, is a cointegrate that contains three replication initiators, two MOB regions and a MPF system. With only five exceptions, the retrieved plasmids contained either a MOB relaxase and/or a MPF system. Besides, only two plasmids smaller than 30 kb, CloDF13 (9.9 kb) and ColEST258 (13.6 kb), were found to encode a FI factor (FinC). Thus, the proteins analyzed here seem to be associated with plasmids transmissible by conjugation, and especially with conjugative plasmids.

## Discussion

The MOB_F11_/IncW plasmid family is formed by a set of broad host range conjugative plasmids capable of transferring antibiotic resistance genes to distantly related bacteria (Mazodier and Davies, 1991; Revilla et al., 2008; Garcillan-Barcia et al., 2009; Fernandez-Lopez et al., 2017). Its relatively simple genetic organization (Revilla et al., 2008; Fernandez-Lopez et al., 2014) and widespread MPF system (Christie et al., 2014; Guglielmini et al., 2014), place it as a suitable prototype group to study plasmid routes for antibiotic resistance propagation, as well as to find new barriers that control plasmid conjugation.

Among 10 different incompatibility groups of conjugative plasmids from clinically representative enterobacteria, four (IncFI, IncI1, IncP-1α, and IncX2) inhibited conjugation of MOB_F11_ plasmids, whereas two (IncP-1α and IncP-1β) were inhibited by them (Table 1). Specifically, plasmids pOX38 (IncFI), R64*drd11* (IncI1), pRL443 (IncP-1α), and R6K (IncX2) repressed R388 conjugation, while IncW plasmids R388 and pIE321 inhibited conjugation of plasmids pRL443 (IncP-1α) and R751 (IncP-1β). The IncFI and IncI1 FI effects on MOB_F11_ plasmids had not been previously reported. The FI effect of IncP-1α plasmids on IncW conjugation was attributed to *fiwA* and *fiwB* FI genes (Fong and Stanisich, 1989). R6K was described as a fertility inhibitor of IncW and IncP plasmids by an unidentified mechanism (Olsen and Shipley, 1975). It also prevented conjugation of IncN plasmids and infection by N-specific bacteriophage IKe. Thus, R6K-encoded FI system might be affecting pilus formation. In contrast, the slight enhancement effect on R388 conjugation caused by pKM101 could be explained by earlier experiments showing that R388 MOB transfer using its own MPF occurs as efficiently as employing pKM101 MPF (Llosa et al., 2003). Inhibition of IncP conjugation by IncW plasmids had also been reported (Olsen and Shipley, 1975), but no inhibitory gene had been identified.

Recently, it was found that Osa and other FI proteins, either homologs to Osa (FiwA of RP4 and ICE1056Fin of ICEhin1056) or unrelated (FipA and PifC), inhibited transfer of T-DNA from *Agrobacterium tumefaciens* to plant cells (Maindola et al., 2014). That study indicated that the same FI factor prevents transfer of more than one conjugative system. Thus, one single plasmid could compete with several unrelated conjugative systems at the same time. Since the FI factor *pifC*, encoded in F and R64, was involved in the inhibition of T-DNA transfer to plants (Maindola et al., 2014), and RP4 conjugation (Miller et al., 1985), we tested *pifC* as a potential factor responsible for IncW repression. The expression of *pifC* in donor bacteria inhibited conjugation of MOB_F11_ plasmids R388 or pMBUI4 (Figure 1), in addition to IncP plasmids pRL443 or R751, as expected (Miller et al., 1985). These results disagree with previous data, in which expression of F-encoded *pifC* affected RP4 but not R388 conjugation (Santini and Stanisich, 1998).

Once established that the factor required for IncP and IncW FI by IncFI and IncI1 rival plasmids was PifC, the next step was to identify the target. A variety of evidences indicate that the coupling protein, the conjugative component involved in linking DNA processing and transport, is the target of the FI system. First, mobilization of plasmid CloDF13 by R388 was unaffected by PifC, whereas mobilization of ColE1 and RSF1010 was inhibited (Figure 2). Equivalent results were obtained in previous experiments with plasmid RP4 (Santini and Stanisich, 1998). Second, RSF1010 mobilization by pKM101 was resistant to PifC inhibition, confirming TrwB coupling protein as target. Third, the overexpression of *trwB* in the presence of pOX38 significantly improved R388 conjugation (Figure 3). Although significant, the increase in transfer observed when additional copies of TrwB are present in the cell is limited. The absence of total recovery of conjugation frequency by *trwB* overexpression might be explained if the target of PifC is the interface between two conjugative components acting together, not only TrwB.

The effect of IncW conjugative systems on fertility of IncP plasmids had been observed before, but the responsible gene was unknown. Our observations indicate that *osa*, the inhibitor of T-DNA-mediated plant oncogenicity, is the actual responsible for IncP-1 FI. This conclusion is based on, first, the absence of inhibitory effect when employing IncW plasmids variants lacking *osa* gene as co-resident plasmids (Figure 4A), and second, the inhibition of transfer caused by Osa expression in donor cells (Figure 4B). Similarly to PifC, we investigated the effect of Osa in different conjugative and mobilizable systems to allow the identification of its target during plasmid transfer. The results using mobilizable plasmids that rely on different coupling proteins to be transferred were similar to those obtained with PifC (Figure 4C), confirming the coupling protein as the target of both FI factors.

Our search for FI factors in the NCBI plasmid database revealed their presence in just a small fraction of the completely-sequenced plasmids. FI factors belong to different protein families and are encoded mainly in conjugative plasmids. Plasmid acquisition of these FI factors seems to be recent evolutionary events, as indicated by the facts that (i) conserved plasmid backbones not always encode the same FI factors and (ii) the distribution pattern of a particular factor is not coherent with plasmid backbone phylogenies.

**Figure 6:**
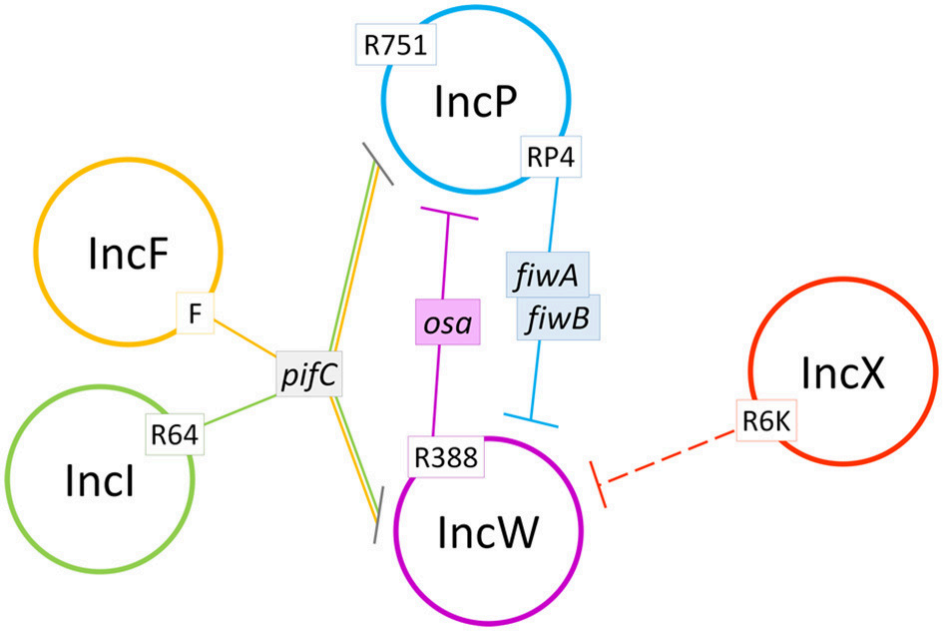
Schematic summary of the plasmid interactions observed in this study. Plasmid incompatibility groups are represented by colored circles. Continuous lines show identified fertility inhibition systems from plasmids in white boxes. Dashed lines show fertility inhibition systems caused by unidentified genes from plasmids in white boxes.

In summary, this work sheds light on the FI interaction network of conjugative plasmids by using the IncW group of broad host range plasmids as a prototype (Figure 6). As shown, FI factors have strong effects on the outcome of inter-plasmid competition for horizontal spread. Specifically, the *pifC* gene of IncF and IncI plasmids, present in a high proportion of *E. coli* clinical and environmental isolates (de Toro et al., 2014), might be at least partially responsible for the low abundance of IncW or IncP plasmids in these populations. FI factors are just one of the many factors that affect plasmid-plasmid interactions (Getino and de la Cruz, 2017). It is important to study these interactions to understand how plasmids, and their attached antimicrobial resistance genes, disseminate in the environment. In fact, FI factors themselves might be useful as actual weapons against the spread of conjugative plasmids, given their wide range of potential target plasmids.

## Author contributions

MG and FdlC conceived the study. MG, MPG-B, and FdlC designed experiments. MG, MPG-B, and CP-G performed experiments. MG, MPG-B, CP-G, and FdlC interpreted data. MG, MPG-B, and FdlC wrote the manuscript.

### Conflict of interest statement

The authors declare that the research was conducted in the absence of any commercial or financial relationships that could be construed as a potential conflict of interest.

## Supplementary material

Supplementary Table 1 List of primers used in this study.

Supplementary Table 2 List of plasmids encoding FI factors.
