# Supplementary material for: PifC and Osa, Plasmid Weapons against Rival Conjugative Coupling Proteins
Source: Front Microbiol. 2017 Nov 16;8:2260. doi: 10.3389/fmicb.2017.02260 (PMC5696584; doi:10.3389/fmicb.2017.02260)
Supplement: Supplementary Table 1 — List of primers used in this study. [file Table1.DOCX]

**Supplementary Table 1**. List of primers used in this study.

| **Primer name** | **Sequence (5’ – 3’)** |
| --- | --- |
| F*Kpn*ITrwB | CATCAGGTACCTTTAAGAAGGAGATATACATATGCATCCAGACGATCAAAGAAAG |
| R*Hin*dIIITrwB | AACAGCCAAGCTTTTAGATAGTCCCCTCAACAAAGGC |
| F*Kpn*IPifC | AGCTCGGTACCTTTAAGAAGGAGATATACATATGATGCTAAGCCAGCTTAACCTG |
| R*Hin*dIIIPifC | ACAGCCAAGCTTTTACAGATCTCCGTACAGGCAGC |
| F*Kpn*IOsa | CATCAGGTACCTTTAAGAAGGAGATATACATATGCTGATGTTGCTACGGCGGCGG |
| R*Hin*dIIIOsa | AACAGCCAAGCTTGATCTTCCTGCATTGCTCACGCAG |
